# Effects of antiepileptic drugs on sleep architecture parameters in adults

**DOI:** 10.5935/1984-0063.20220045

**Published:** 2022

**Authors:** Bruno Miguel Santos Carvalho, João Chaves, António Martins da Silva

**Affiliations:** 1 University of Porto, Abel Salazar Institute of Biomedical Sciences - Porto - Portugal.; 2 Centro Hospitalar do Porto, Serviço de Neurologia, Departamento Neurociências, Hospital de Santo António - Porto - Portugal.; 3 University of Porto, UMIB - Unidade Multidisciplinar de Investigação Biomédica, Instituto de Ciências Biomédicas Abel Salazar, Universidade do Porto - Porto - Portugal.; 4 Centro Hospitalar do Porto, Serviço de Neurofisiologia, Departamento de Neurociências, Hospital de Santo António - Porto - Portugal.

**Keywords:** Polysomnography, Anticonvulsants, Sleep

## Abstract

**Objectives:**

Physiological and restorative sleep is fundamental for physical and mental well-being. Polysomnography parameters are objective methods to access sleep structure. Antiepileptic drugs (AEDs) are a group of drugs whose interference in the sleep structure is still not well known, especially in what concern the new ones. We did a systematic review of the literature to compare the effect of classic and newer AEDs on sleep architecture.

**Material and Methods:**

A search was performed in PubMed and Scopus, using keywords “sleep” and “antiepileptics”, and each AED combined with “sleep”. Only studies concerning objective measures were selected.

**Results:**

63 articles were included, only 21 were randomized, controlled and double-blinded. Studies not only in epilepsy, but also in restless leg syndrome, bruxism, insomnia, fibromyalgia and obstructive sleep apnea were found. Among classic AEDs, carbamazepine has a negative effect on sleep while phenobarbitone has a slightly dose-dependent interference and is also the only one to reduce N3 stage. Valproic acid has little to no effect while clobazam and clonazepam have a positive effect. No conclusion can be drawn about phenytoin. All of them reduce REM stage. In the newer AEDs group gabapentine, lamotrigine, perampanel, pregabaline and tiagabine increase N3 sleep in best evidence. Lacosamide and zonisamide appear to be innocent while levetiracetam reduces REM sleep.

**Conclusion:**

Studies found used different methodologies not always addressing the analysis on the same parameters. In spite of these, newer AEDs have less effects on sleep structure when compared with classic AEDs but furthermore robust evidence is needed.

## INTRODUCTION

A good night of sleep is recognized as essential to optimal physical and mental health. The impact of sleep in the immune system functioning and cognition has already been described^1^. A recommendation of the American Academy of Sleep Medicine (AASM) and the Sleep Research Society is, for adults up to 60 years of age, to sleep seven or more hours on a regular basis^2^. Sleep impairment may lead to excessive daytime sleepiness (EDS), with drowsiness occurring in situations in which an individual is expected to be alert and vigilant^1^. Concomitantly, EDS can also be seen when the total sleep time (TST) is within normal range, particularly when there is a sleep fragmentation, defined by recurrent arousals and/or stage shifts^3^. Early detection of sleep disorders is important, mainly due to its impact on general public health, contributing to an overall increase in the population’s morbidity, mortality and decreased work productivity. A correct diagnosis and management are usually possible and may improve health and quality of life of patient with sleep disorders^2,4^.

Sleep disruption can be suspected by assessing selfreported sleepiness quantification scales, such as the extensively used Epworth sleepiness scale or the Stanford sleepiness scale. These two subjective methods are considered to be more inaccurate in their correlation with sleep structure and, therefore, objective methods are preferred^1^. To the date, traditional in-lab polysomnography (PSG) is still the gold standard for objective assessment of sleep structure^4-6^. Actigraphy, maintenance of wakefulness test (MWT) and multiple sleep latency test (MSLT) are also objective methods used for determining the treatment effects on sleeping and daytime sleepiness^2,4-6^.

Through a standardized PSG exam, sleep can be divided into two main cycles: rapid-eye movement (REM) and non-rapid eye movement (NREM) sleep cycles. Up to 2007, Rechshaffen and Kales classification stratified NREM sleep in four stages NREM - S1, S2, S3, and S4^7,8^. Because frequent difficulties to classify and split S3 from S4 a task force from the American Academy of Sleep Medicine adopt the proposal from Silber et al. (2007)^10^ and merge S3 and S4 into a unique stage NREM N3 that define the slow wave sleep^9,10^. In addition to the sleep stages, there are other sleep parameters that can also be assessed such as arousals, sleep efficiency (SE), sleep latency (SL), awakenings and wake after sleep onset (WASO)^3^. Although most changes in sleep structure caused by AEDs have been considered negatives, there are some which are seen as improvements, such as a decreased number of arousals and/or N1 stage and an increased SWS or REM sleep^6^. During NREM1 to NREM3 some individuals present a prolonged cyclic alternation of high voltage slow waves and low-voltage irregular activity. This can be described as cyclic alternating patterns (CAP) and this sleep microstructure alteration has been considered an EEG marker of unstable sleep^11^.

Antiepileptic drugs (AEDs) are a heterogeneous class of drugs used worldwide, mainly in epilepsy as antiseizure medication. These can be classified according to their mechanism of action or in classic AEDs and newer ones. In theory, the new AEDs have fewer side effects. AEDs are not used exclusively in epilepsy, in fact, its use has been expanded to treat other pathologies, such as neuropathic pain^12^, fibromyalgia^13^, mood disorders^14^, bruxism^15^, restless leg syndrome (RLS)^14^ or periodic limb movement disorder^16^, pathologies that disturb sleep and sleep structure. The current knowledge is that all AEDs can influence sleep architecture, but there may be a positive or a negative impact caused by the drug *per se* or by the type of epilepsy^17^. Sleep disturbances can also exacerbate the drowsiness and memory dysfunction, common in patients with epilepsy (PWE), and be a factor itself of difficulty in controlling seizures^18,19^. According to the reported literature, most of the classic AEDs have some detrimental effect on sleep. New AEDs do not seem to affect nocturnal sleep in the same way but the literature is scarce in relation to objective analysis of sleep^20^. However, sleep disturbances may be not only related with the AEDs but also may be a consequence of seizures itself^21^. Polytherapy is also another independent risk factor for excessive daytime somnolence (EDS), with patients taking more than one AED being at a higher risk than those on monotherapy^22,23^. But again, the reason for such EDS is not always a linear relationship found between the pharmacological AEDs characteristics.

It is known that sleep disturbances are associated with decreased quality of life in chronic diseases such as epilepsy^5^. In PWE, a group with a particularly high incidence of sleep-related complaints, sleep disorders represent an increased risk in seizures control^4,6^. PWE have a higher frequency of arousals, awakenings and phase shifts compared to the general population, even when they are controlled or without AEDs treatment^19^. These three transitional phase events create conditions of brain network instability and increase ictal and interictal epileptic phenomena, which can also alter the sleep-waking cycle and sleep architecture^6,24^. New evidence in this field is a much needed area of research as the most recent systematic review on the impact of epilepsy per se in sleep parameter by Sudbrack-Oliveira et al. (2019)^25^ only established conclusions about an increase in WASO in temporal lobe epilepsy. Precluding conclusions on other subgroups of PWE due to study heterogenicity.

Despite their extensive use, objective influence of AEDs on sleep is not yet full perceived^21^. Since drowsiness is one of the most common reported AEDs side effects, some direct influence may indeed be suggested^6^. Maestri et al. (2013)^26^ in the novo untreated PWE have already concluded that there were no significant differences regarding sleep parameters compared to the control group and, thus, EDS may be due to a factor other than epilepsy *per se.* Larger studies are needed to confirm this conclusion, but this is a challenging task, as testing the long-term effect of most AEDs in individuals without epilepsy is unethical, preventing the formation of larger control groups, and it is dangerous and incorrect to switch off the AEDs in PWE just to control these confounding factors^27^. There is also not enough research with focus on non-benzodiazepine drugs to conclude its usefulness and safety in insomnia’s treatment, because designing large trials without bias is complicated and expensive^28^.

The aim of this review is to collect all the evidence available in common databases about the direct influence of the newer AEDs on sleep architecture, as well as to compare such parameters with the classic AEDs. With this, we intend to understand better the effect of AEDs not only in the treatment of epilepsy but also in other pathologies in which AEDs are used.

## MATERIAL AND METHODS

Our research was carried out, from March 1^st^ to April 1^st^ 2020, in PubMed and Scopus databases, using the MESH-terms “antiepileptic drugs” and “sleep” as keywords. Only clinical trials with individuals treated with acute or chronic AEDs and written in English were selected.

**Figure 1:**
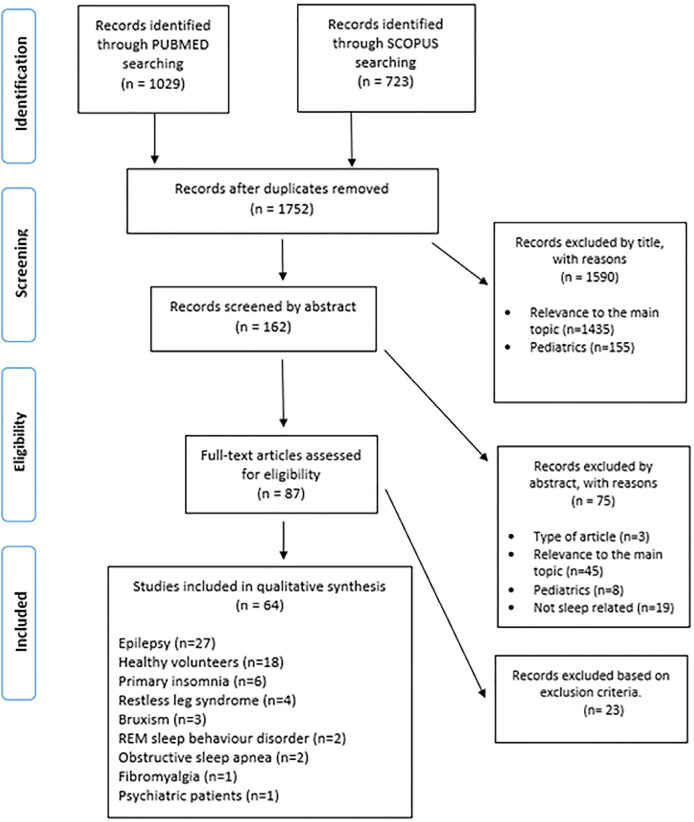
Diagram with screening selection methodology. Adapted from PRISMA. From: Moher et al. (2009)^29^.

In this review, we included trials studying the effects of AEDs in subjects not only with epilepsy, but also with other disorders and in healthy adult volunteers. Trials involving children or concerning non-pharmacological treatments of epilepsy, such as vagus nerve stimulation or surgery, were excluded. The search conducted in the aforementioned databases also combined individual AED names with “sleep” (for example “carbamazepine AND sleep”). After removing all duplicated articles, the results were added to the first search, as shown in Figure 1. For analysis, we divided AEDs in classic and newer ones. We considered classic AED’s the older ones, that came out before the early eighties. The classic AED searched were carbamazepine, clobazam, clonazepam, ethosuximide, phenobarbital, phenytoin and valproic acid. The newer AED searched included brivaracetam, eslicarbazepine acetate, felbamate, gabapentin, lacosamide, lamotrigine, levetiracetam, oxcarbazepine, perampanel, pregabalin, rufinamide, stiripentol, sulthiame, tiagabine, topiramate, vigabatrin and zonisamide. Multiple articles concerning BZDs were obtained, however, in our review, we only focused on clonazepam and clobazam, because these are also used as AEDs. Other trials including BZD were excluded.

The articles selected for qualitative review included only the ones that assessed objective sleep outcomes. Articles concerning only subjective sleep outcomes were excluded. The objective sleep outcomes considered were the PSG measurements previously described, such as sleep stages and other sleep parameters. Articles reporting MLST results were also included, as this is a validated objective parameter. In this review, studies considering the old classification of stages N3 and N4 were reported here as slow wave sleep (SWS). The class of evidence was based on the recommendations from American Academy of Neurology^30^.

## RESULTS

After the bibliographic search, summarized in Figure 1, sixty-four articles were selected to qualitative analysis. Of these, only ten articles were published in the last 5 years. Eighteen focused only on classic AEDs, Thirty-nine focused exclusively on one of the newer AEDs and seven assessed more than one AED simultaneously. Tables 1, 2 and 3 summarize the significant results reported in the articles selected for analysis.

**Table 1:** Summary of studies on classic antiepileptic drugs (n=18).

	Study (date and reference)	Type of study (class of evidence)	N	Population	Type of therapy	Dose (per day)	Comparison	Significative PSG results (mean ± SD)
**Carbamazepine**	**Yang et al. (1989)^32^**	Prospective, non- randomized, non- blind (class III)	7	Healthy male volunteers	Acute monotherapy	Mean plasma range 11.8+1.1 |ig/ml 200-700mg/day	NA	**•fSWS (%):** in CBZ (35.3+10.8) vs. baseline (16.8+9.7)** **REM(%):** in CBZ (19.5+4.3) vs. baseline (22.8+4.3)**
	**Manni et al. (1990)^33^**	Observational, non- randomized, non- blind (class III)	14	Focal epilepsy	Chronic monotherapy	15-20mg/kg	Healthy controls (n=11)	**Patients with poor seizure control (mean 2.7 seizures per month; n=6): REM(%):** in CBZ (16.8+5.6) vs. controls (29.6+5)* **^REM latency (min):** in CBZ (140.4+32.5) vs. controls (75.1+16.2)* **•fWASO:** in CBZ (42.5+17.4) vs. controls (13.4+10.8)* **No. of awakenings:** in CBZ (10.3+4.0) vs. controls (2.6+1.7)* **No. of shifts to stage N1:** in CBZ (13.0+6.8) vs. controls (3.7+1.6)*No. of stage shifts: in CBZ (47.8+9.8) vs. controls (21.0+4.5)*Patients with complete seizure control (n=8): **REM latency (min):** in CBZ (106.4+24.3) vs. controls (75.1+16.2)*
								**No. of awakenings:** in CBZ (9.5+5.6) vs. controls (2.6+1.7)* **‘TNo. of shifts to stage N1:** in CBZ (10.0+4.1) vs. controls (3.7+1.6)* **No. of stage shifts:** in CBZ (40.0+10.5) vs. controls (21.0+4.5)* There were no significative differences between the groups.
	**Riemman et al. (1993)^36^**	Prospective, non- randomized, non- blind (class III)	12	Healthy male volunteers	Acute monotherapy	400mg/day	NA	**After 5-day course of CBZ:** **•fSE (%):** in CBZ (95.2+1.6) vs. baseline (91.1+4.0)** * **N2 latency (min):** in CBZ (9.9+6.3) vs. baseline (23.8+13.7)** **Wake time (%):** in CBZ (1.6+1.4) vs. baseline (3.5+2.9)* **•fSWS (%):** in CBZ (13.2+7.0) vs. baseline (6.8+5.3)** **Total REM density (%):** in CBZ (16.6+5.6) vs. baseline (21.8+5.1)*
	**Gigli et al.** **(1997)^34^**	Prospective non- randomized, single- blinded (class III)	16 (n=7 patients)	Temporal lobe epilepsy	Acute or chronic monotherapy	Initial dose: 400mg Following: 800mg	Healthy subjects	Acute CBZ in controls: **“TStage shifts:** in acute CBZ (211.9+78.7) vs. baseline (176.9+56.4)** * Acute CBZ in TLE: **REM(%):** in acute CBZ (14.1+3.9) vs. baseline (17.6+4.8)** **No. of entries in REM:** in acute CBZ (44.9+12.3) vs. baseline (31.6+5.8)** No significative changes in chronic CBZ
	**Gann et al.** **(1994)^35^**	Prospective, nonrandomized, uncontrolled	12	Healthy volunteers	Acute monotherapy	400mg/day	NA	**At night 5:** **•fSE(%):** in CBZ (95.2+1.6) vs. baseline (91.1+4.0)** * **Stage N2 latency (min):** in CBZ (9.9+6.3) vs. baseline (23.8+13.7)** **•fSWS(%):** in CBZ (13.2+7.0) vs. baseline (6.8+5.3)**
		(class III)						**Total REM-density (%):** in CBZ (16.6+5.6) vs. baseline (21.8+5.1)*
	**De la Fuente et al. (2002)^31^**	Randomized, single blinded (class II)	20	Borderline personality disorder	Acute monotherapy	400- 800mg/day	Placebo (n=10)	**At baseline:** **Stage N1:** in CBZ (5.67+2.07) vs. placebo (9.63+3.38)** **After treatment:** **•fStage N3(%):** in CBZ (4.95+2.39) vs. baseline (2.38+2.63)** **•fSWS(%):** in CBZ (11.81+10.63) vs. baseline (4.14+5.17)**
	**Nayak et al. (2016)^39^**	Cross-sectional case- control (class III)	40	Temporallobe epilepsy	Drug-naive (n=20) CBZ monotherapy (n=20)	NA	Healthy controls (n=40)	**•fTST (h):** in CBZ (8.50+0.58) vs. controls (7.48+0.55)** * **SE(%):** in CBZ (76.38+12.23) vs. controls (84.64+9.51)* **Stage N1 (%):** in CBZ (4.50+2.69) vs. controls (8.07+4.22)* **REM arousal index:** in CBZ (9.30+7.39) vs. controls (4.84+6.35)** * No significative differences in controls vs. drug-naive patients
**Clobazam**	**Nicholson et al (1977)^41^**	Prospective, Randomized, double-blind (class I)	6	Healthy adults	Single-dose (10mg or 20mg/day)	10mg, 20mg	Placebo and group with triflubazam	**Clobazam 10mg:** **4/Stage N1(%):** in CLO (4.4+32) vs. placebo (6.6+32)* **4/Sleep onset latency (min):** in CLO (17.7+40) vs. placebo (27.1+40)* **Clobazam 20mg:** **4/Stage N1(%):** in CLO (4.3+32) vs. placebo (6.6+32)* **•fStage N2(%):** in CLO (54.7±8) vs. placebo (49.2±8)*Stage N3(%): in CLO (7.7±19) vs. placebo (11.2±19)**SWS(%): in CLO (16.3+16) vs. placebo (19.7+16)* **Sleep onset latency (min):** in CLO (16.0±40) vs. placebo (27.1±40)*
**Clonazepam**	**Saletu et al. (2010)^45^**	Single-blind, nonrandomized, crossover (class III)	21	Drug-free adults with bruxism	Single-dose (1mg/day)	1mg	Healthy controls and placebo	**No. of awakenings:** in CNZ (8.4±5.3) vs. placebo (13.0±6.2)** **Awakening index (no/h of sleep):** in CNZ (1.2±0.8) vs. placebo (2.4±1.3)** **WASO (min):** in CNZ (17.7±19.2) vs. placebo (54.0±61.0)** **•fTST (min):** in CNZ (419.1±22.6) vs. placebo (372.0±71.3)** **•fSE (%):** in CNZ (93.0±5.0) vs. placebo (83.3±15.9)** **Stage N1 (%):** in CNZ (4.3±3.1) vs. placebo (7.5±3.6)** **•fStage N3(%):** in CNZ (9.0±5.3) vs. placebo (7.1±3.8)** **Stage shift index (n/h of sleep):** in CNZ (17.5±4.4) vs. placebo (21.0±5.1)**
	**Ferri et al. (2013)^42^**	Observational (class III)	57	Idiopathic REM sleep behavior disorder (iREMSBD)	Chronic monotherapy	0.5-1.0mg	iREMSBD taking bedtime clonazepam (n=15) Control: iREMSBD	**Comparison between the two groups at baseline:** **Stage shifts (n/hour):** in in CNZ (12.8±4.7) vs. control (16.5±6.59)* **•fSE (%):** in CNZ (83.6±6.99) vs. control (76.0±13.08)* **WASO (%):** in CNZ (10.1±6.19) vs. control (17.9±9.84)** **Stage N1 (%):** in CNZ (6.6±3.06) vs. control (9.0±4.08)* **•fStage N2 (%):** in CNZ (46.2±10.88) vs. control (38.7±8.81)**
							drug-free (n=42)	There were no statistically significant differences in the first follow-up (2.6+1.08 years) of patients taking CNZ (n=13).
	**Sakai et al. (2016)^43^**	Randomized, double blind, crossover (class I)	19	Primary sleep bruxism	Acute monotherapy, crossover with clonidine	1mg	Placebo	No significative differences in sleep parameters in CNZ vs. placebo.
	**Ferri et al. (2017)^46^**	Observational (class III)	64	Idiopathic REM sleep behavior disorder (iREMSBD)	Chronic monotherapy or acute monotherapy	0.5-2mg	Drug-naive (n=29) Chronic CNZ therapy (n=14) Healthy controls (n=21)	**In chronic treatment patients (n=14) vs. drug naïve patients (n=29) Stage N2:** in chronic CNZ (44.6+9.14) vs. drug-naïve patients (38.5+8.53)*In chronic treatment patients (n=14) vs. Controls (n=21) **Awakenings (/hour):** in chronic CNZ (4.0+2.32) vs. controls (7.2+2.9)* **SE (%):** in chronic CNZ (83.5+7.85) vs. controls (68.3+12.55)** **SWS (%):** in chronic CNZ (17.5+6.75) vs. controls (10.4+7.82)** *
**Phenobarbital**	**Prinz et al. (1981)^50^**	Prospective, non- randomized (class III)	5	Healthy volunteers	Acute and chronic monotherapy	100mg	Placebo	**Acute:** significative differences vs. placebo **Chronic (1 month):** **4/Sleep latency (min):** in PB (3.40+0.87) vs. placebo (8.23+2.50) **Stage N4 (%):** in PB (2.95+0.89) vs. placebo (9.36+1.98)
	**Karacan et al. (1981)^49^**	Double-blind, nonrandomized, crossover	24 (males only)	Healthy volunteers	Acute monotherapy	80, 140, 240mg	Placebo	**Results presented in (mean).** 80mg: **4/No. of stage shifts:** in PB night 1 (34.5) vs. placebo (39.4)*
		(class I)						**Stage REM (%):** in PB night 1 (21.9) vs. placebo (25.5)** **•fStage N2(%):** in PB night 1 (61.0) vs. placebo (56.1)* **No. of awakenings:** in PB night 2 (0.9) vs. placebo (1.6)*Latency to stage 0 (min): in PB night 2 (305) vs. placebo (205)*Stage REM (%): in PB night 2 (21.2) vs. placebo (25.4)** * **•fStage N2(%):** in PB night 2 (60.7) vs. placebo (56.6)* 140mg: **4/Stage REM (%):** in PB night 1 (18.9) vs. placebo (25.5)** * **“TStage N2(%):** in PB night 1 (62.1) vs. placebo (56.1)** **4/REM episode duration (min):** in PB night 1 (22.4) vs. placebo (26.3)* **cNo. of awakenings:** in PB night 2 (0.7) vs. placebo (1.6)**Latency to stage 0 (min): in PB night 2 (301) vs. placebo (205)*4/Stage REM (%): in PB night 2 (19.6) vs. placebo (25.4)** *“TStage N2(%): in PB night 2 (63.4) vs. placebo (56.6)** * **4/REM episode duration (min):** in PB night 2 (21.4) vs. placebo (27.9)** * 240mg: **4/No. of stage shifts:** in PB night 1 (32.9)* vs. placebo (39.4)**Latency to REM (min): in PB night 1 (148) vs. placebo (85)** *4/Stage REM (%): in PB night 1 (16.1) vs. placebo (25.5)** *“TStage N2(%): in PB night 1 (66.3) vs. placebo (56.1)** *4/No. of REM episodes: in PB night 1 (3.5) vs. placebo (4.2)** **REM episode duration (min):** in PB night 1 (21.6) vs. placebo (26.3)* **4/No. of awakenings:** in PB night 2 (0.2) vs. placebo (1.6)** *4/No. of stage shifts: in PB night 2 (27.5)* vs. placebo (34.8)**Latency to stage N1 (min): in PB night 2 (389) vs. placebo (205)** *Latency to REM (min): in PB night 2 (171) vs. placebo (98)** *4/Stage REM (%): in PB night 2 (15.8) vs. placebo (25.4)** *“TStage N2(%): in PB night 2 (67.4) vs. placebo (56.6)** * **4/No. of REM episodes:** in PB night 2 (3.2) vs. placebo (4.2)** * **4/REM episode duration (min):** in PB night 2 (21.8) vs. placebo (27.9)**
**Phenytoin**	**Roder-Wanner et al. (1987)^53^**	Prospective longitudinal, non- blind (class III)	31	Patients with new-onset epilepsy	Acute monotherapy	100mg	Placebo	Acute 100mg PHE in whole group (n=31): **“TStage N4 (%):** in PHE (41.5+32.4) vs. placebo (29.1+27.3)* Acute 100mg PHE in group with generalized epilepsy (n=9): **“TStage N4 (%):** in PHE (53.0+34.5) vs. placebo (36.8+29.5)** Acute 100mg PHE in group with focal epilepsy (n=8): **REM(%):** in PHE (17.1+ 8.4) vs. placebo (26.7+12.9)* **After 4-6 weeks of PHE (check figure in paper for values):** **xVSleep latency (min):** in PHE vs. baseline* **Stage N1 (%):** in PHE vs. baseline* **Stage N2 (%):** in PHE vs. baseline* **•fSWS (%):** in PHE vs. baseline* **REM(%):** in PHE vs. baseline*
**Valproic acid**	**Harding et al. (1985)^56^**	Prospective, single- blind (class III)	10	Healthy adults	Acute monotherapy	2 days placebo, 2 days placebo + 500mg VPA, 14 days 1,000mg VPA	Placebo	**Visual evocked potential (VEP) and EEG sleep recording:** **- No significative differences in VEP On sleep EEG:** (no values presented) **REM:** in high dose VPA vs. placebo** *REM: in high dose VPA vs. low dose** *REM: in low dose VPA vs. withdrawal** * *TSWS: in high dose VPA vs. placebo* *TSWS: in high dose VPA vs. low dose**
	**Eisenser et al. (2004)^54^**	Randomized, placebo-controlled, double-blind, cross- over study (class I)	20	Idiopathic RLS	Acute monotherapy	300mg-SR In the first 2 days followed by 600mg-SR	Placebo and crossover with Levodopa	Latency to stage N2: In VPA-SR (40.6±28.8) vs. placebo (29.7±26.8);*
	**Nayak et al. (2016)^57^**	Case-control cross- sectional study (class III)	40	Juvenile myoclonic epilepsy	N=20 drug naïve N=20 on VPA		Healthy controls	**REM** in VPA (28.76 ± 12.23) vs. drug naïve patients (13.83±5.91) vs. controls (16.26±4.01)** *

Abbreviations: CBZ = Carbamazepine; CLO = Clobazam; CNZ = Clonazepam; PB = Phenobarbital; VPA = Valproic acid;

* *p*<0,05;

** *p*>0,01;

*** *p*<0,001.

**Table 2:** Summary of studies on new antiepileptic drugs (n=39).

	Authors	Type of study (class of evidence)	N	Population	Type of therapy (AED dose)	Dose (per day)	Comparison	Significative PSG results (mean ± SD)
**Felbamate**	**Marciani et al. (1998)^58^**	Case report (class IV)	1	Lennox-Gastaut syndrome	Acute add-on therapy	1,800mg	NA	**After 4 months:** **4/Phase shifts:** in FEL (21.4) vs. baseline (41.8) **No. of Awakenings:** in FEL (17) vs. baseline (43) **4/Stage N1 (%):** in FEL (39.9) vs. baseline (54.5) **WASO(%):** in FEL (16) vs. baseline (18.6)
**Gabapentin**	**Rao et al. (1988)^62^**	Double-blind, placebo- controlled, randomized crossover (class I)	6	Healthy adults	Acute monotherapy	1st day: placebo 2nd day: GBP 300mg 3rd day: GBP 600mg	Placebo	**Deep sleep latency:** in GBP was decreased to 70% vs. controls* **SWS:** was increased to 120% in GBP vs. controls*
	**Placidi et al (2000)^24^**	Prospective, non- randomized (class III)	18	Drug resistant epilepsy focal cryptogenic (n=12) Focal symptomatic (n=6)	Acute add-on therapy	1,800-2,400mg/day	NA	**4-months vs. baseline:** **Stage N1:** in GBP (9.7±4.2) vs. baseline (14.4±3.2)** * **•fSWS(%):** in GBP (17.1±6.5) vs. baseline (14.4±5.0)* **•fREM(%):** in GBP (14.7+5.5) vs. baseline (11.1+3.1)** * **No. of awakenings:** in GBP (13.9±7.4) vs. baseline (21.6±11.0)** *
	**Foldvary- Schaefer et al. (2002)^59^**	Prospective, non- randomized, open label (class III)	19	Healthy adults	Acute monotherapy	300-1,800mg	Healthy controls (n=9)	**GBP group (n=10):** **•fSWS(%):** in GBP (13.0+0.07) vs. baseline (8.0±0.05)**
	**Lo et al. (2010)^64^**	Open-label (class IV)	18	Primary insomnia	Acute monotherapy	Mean dose = 540mg (200 900mg)	NA	**After 4 weeks of GBP treatment:** **•fSE (%):** in GBP (87.17+11.37) vs. baseline (80.00±14.79)* **- WASO (min):** in GBP (7.84+9.92) vs. placebo (16.45±14.78)* **•fSWS (%):** in GBP (17.68+9.88) vs. baseline (10.47±9.86)** *
	**Madani et al.** **(2012)^15^**	Single-blind, randomized clinical trial (class II)	20 GBP group (n=10)	PSG documented sleep bruxism	Acute monotherapy	100mg during 3 nights 200mg the next 3 nights 300mg from the 6th week to 2 months	Stabilization splint	**Before treatment:** **•fstage N3 (%)** in GBP (18.1+2.6) vs. splint group (15.7±2.5) at baseline **Versus baseline:** **•fTST (min):** in GBP (441.7+45.0) vs. baseline (382±40.9) **•NREM Sleep latency (min):** in GBP (10.9+9.9) vs. baseline (24.2±14.1) **f SWS (%):** in GBP (20.9+4.0) vs. baseline (18.1±2.6) **f SE (%):** in GBP (93.1+3.9) vs. baseline (86.3±4.8)
	**Furey et al. (2014)^61^**	5-hour sleep advance, randomized, double-blind, placebo-controlled, multicenter (class I)	237	Adults with sleep complaints	Acute monotherapy	250mg	Placebo (n=115)	Gabapentin (n=122) Day 1: **•fTST (min):** in GBP (347.6±7.5) vs. placebo (283.9±9.8)** *WASO (min): in GBP (107.0±7.3) vs. placebo (149.1±9.6)**Stage N1 (%): in GBP (12.3+0.6) vs. placebo (15.7+1.1)* Day 28: **•fTST (min):** **WASO (min):** in GBP (113.6±8.1) vs. placebo (152.3±9.3)** **REM (%):** in GBP (15.6±0.5) vs. placebo (13.6±0.6)*
	**Rosenberg et al. (2014)^60^**	Randomised, double-blind, multicenter clinical trial (class I)	Placebo (n=124) 250mg (n=124) 500mg (n=125)	Adults with sleep complaints	Acute monotherapy	250 or 500mg groups	Placebo	**•fTST (min):** in 500mg (378.7±7.3)** * or 250mg (356.5±7.3)** * vs. placebo (311.4±8.4) **WASO (min):** in 500mg (73.2±5.8)** * or 250mg (100.7±6.6)** * vs. placebo (135.7±7.0) **Stage N1 (min)**: in 500mg (10.8±0.7)** * or 250mg (11.8±0.7)** * vs. placebo (15.1±1.0) **•fSWS (%)** in 500mg (17.0±1.1)** * or 250mg (15.4±1.0)* vs. placebo (12.6±0.9)
	**Piovezan et al. (2017)^63^**	double-blind, randomized, placebo- controlled crossover pilot study (class II)	8	Healthy men (>60 years old)	Acute monotherapy	300mg	Placebo	No significative changes in objective sleep parameters were found.
**Lacosamide**	**Hudson et al. (2015)^67^**	Multicenter, interventional, open-label study (class III)	25	Healthy adults	Monotherapy	300mg	NA	No significative changes in objective sleep parameters were found.
	**Foldvary- Schaefer et al. (2017)^66^**	Phase IV randomized, controlled, double blind, single center trial (class I)	52	Focal epilepsy	Acute adjunctive	400mg	Placebo (n=10)	**Comparison between LAC (n=42) and placebo (n=10) at baseline Arousal Index:** in LAC [6.1(4.9-8.4)] vs. placebo [10.3 (7.0-13.7)]*Comparison between LAC (n=35) and baseline at follow-up Arousal Index: in LAC-5.0 [-14.0, 3.0] vs. baseline* values expressed in median (P25-P75)
**Lamotrigine**	**Placidi et al. (2000)^20^**	Prospective, uncontrolled, non-randomized (class III)	13	Drug-resistant	Acute adjunctive	200mg	NA	**REM (%):** in LTG (13.3+6.0) vs. baseline (8.5±4.3)** **SWS (%):** in LTG (12.4+6.0) vs. baseline (17.8±6.1)* **No. of entries in REM:** in LTG (36.0±27.0) vs. baseline (16.0±12.0)* **No. of phase shifts:** in LTG (38.0±11) vs. baseline (32.0±11.0)*
	**Foldvary et al. (2001)^68^**	Prospective, uncontrolled, non-randomized (class III)	10	Focal epilepsy	Acute adjunctive Chronic monotherapy: CBZ (n=7); PHT (n=3)	400mg	NA	**•fStage N2 (%):** in LTG (57.5) vs. baseline (50.9)* **SWS (%):** in LTG (13.0) vs. baseline (20.2)* Authors did not present standard deviation. Results in (mean).
**Levetiracetam**	**Bell et al. (2002)^6^**	Double-blind, randomized crossover (class I)	N=12 volunteers N=16 patients	Study 1: healthy volunteers; Study 2: focal epilepsy on carbamazepine monotherapy	Adjunctive	1,000mg	Placebo	Study 1 (n=12): **•fStage N2 (min):** on LEV (270.0±34.2) vs. placebo (235.0±45.5) **•fREM latency (min):** on LEV (121.3±70.0) vs. placebo (69.8±) Study 2 (n=16): **•fStage N2 (min):** on LEV (231.0+33.6) vs. placebo (174.0+21.9) **Stage N4 (min):** on LEV (58.0±30.1) vs. placebo (72.0±27.2)
	**Bazil et al. (2005)^18^**	Randomised prospective (class II)	12	Healthy	Monotherapy	3 days 500mg, 3 days 1,000mg, 3 days 1,500mg and 3 days 2,000mg	Placebo	**No. of awakenings** in follow-up LVT (17.00+1.60) vs. placebo (8.00+1.80)*
	**Cicolin et al. (2006)^71^**	Double-blind crossover (class I)	14	Healthy volunteers	Acute monotherapy	Up to 2,000mg/day	Placebo	**•fTST (min):** in LEV (391.44+30.58) vs. placebo (363.72+40.04)** **•fSE (%):** in LEV (92.78+1.10) vs. placebo (90.61+1.47)** **•fStage N2 (min):** in LEV (253.09+23.61) vs. placebo (228.22+30.02)** * **•fStage N4 (min):** in LEV (23.18+3.58) vs. placebo (16.28+3.91)** * **WASO (min):** in LEV (12.71+1.44) vs. placebo (18.00+4.76)** **•fStage N2 (%):** in LEV (64.67+3.66) vs. placebo (62.71+3.83)** * **•fStage N4 (%):** in LEV (5.92+0.77) vs. placebo (4.47+0.89)** *REM(%): in LEV (14.99+2.59) vs. placebo (17.55+3.59)**WASO (%): in LEV (3.26+0.39) vs. placebo (4.97+1.24)** **Stage shifts (No.):** in LEV (66.00+4.42) vs. placebo (73.50+7.01)** * **MSLT:** No significant changes were detected.
	**Yilmaz et al. (2007)^72^**		22	Complex focal epilepsy	Chronic monotherapy (n=10); Chronic add-on therapy (n=12)	2,000mg/day	Control group (n=20)	**Measured by actigraphy and MWT After 3 weeks of LEV monotherapy:** **xVTotal activity score (no./night):** in LEV (7779+7861) vs. baseline (9840+7323)**Daytime napping episodes (no./day): in LEV (3.45+1.80) vs. baseline (2.44+0.42)**“TTotal nap duration (min/day): in LEV (38.00+8.23) vs. baseline (23.00+3.90)**
	**Zhou et al. (2012)^27^**	Prospective, nonrandomized (class III) Observational prospective (class III)	20	Focal epilepsy without sleep disorders	Monotherapy (n=4) Adjunctive (n=6)	1,000mg/day	Healthy volunteer controls (n=10)	**After 3 weeks of LEV as add-on:** **Total activity score (number/night):** in LEV (5980+8200) vs. baseline (9840+7323)*Daytime napping episodes (no./day): in LEV (6.70+1.20) vs. baseline (2.44+0.42)*“TTotal nap duration (min/day): in LEV (59.00+9.32) vs. baseline (23.00+3.90)* **1 week LEV (n=10) vs. controls (n=10):** **REM latency (min):** in LEV (176.94+94.43) vs. controls (98.75+60.97)* **1 week LEV (n=10) vs. Baseline (n=10):** **REM (min):** in LEV (59.55+33.27) vs. baseline (71.45+37.12)* **REM (%):** in LEV (13.29+6.80) vs. baseline (15.67+7.26)* **MSLT:** No significant changes were detected.
**Perampanel**	**Gonzalez- Cuevas et al. (2016)^73^**	Prospective, non- interventional (class III)	10	Focal epilepsy	Adjunctive	4mg	NA	**MWT (n=10):** No statistically significant differences were found in objective sleep parameters.
	**Garcia- Borreguero et al. (2017)^74^**		20	Idiopathic RLS	Monotherapy	2mg (increased to 4mg in week 4 if necessary)	NA	**•fTST (min):** in PMP (364.64+44.42) vs. baseline (329.85+39.30)*Sleep latency (min): in PMP (20.68+5.10) vs. baseline (32.60+13.10)*Sleep efficiency(%): in PMP (85.64+5.22) vs. baseline (73.02+7.43)** *
		Prospective, open trial (class III)				Mean: 3.8mg/day		**WASO (min):** in PMP (39.99+19.41) vs. baseline (89.25±26.12)** * **•fArousal Index:** in PMP (25.06+6.33) vs. baseline (37.29+12.58)** **Stage N1 (min)**: in PMP (57.59+19.46) vs. baseline (73.10+19.86)* **•f Stage N3 (min)**: in PMP (52.00+10.94) vs. baseline (29.45+18.40)** **Stage N1 (%)**: in PMP (16.13+5.66) vs. baseline (22.40+6.44)** **•fStage N3 (%)**: in PMP (14.40+3.04) vs. baseline (8.90+5.45)** **REM latency (min):** in PMP (86.55+20.72) vs. baseline (115.20+36.65)** **PLM:** in PMP (2.53+0.85) vs. baseline (3.03+0.86)* **PLM index:** in PMP (4.36+2.00) vs. baseline (27.76+6.75)** *
	**Rocamora et al. (2020)^75^**	Prospective open- label pilot study (class III)	17	Refractory epilepsy	Adjunctive	2mg/day increased by 2mg after 2 weeks and then monthly until the target dose of 4-8mg/ day.	NA	**In all patients group (n=17)** **•fTST (min):** in PMP (399.8 + 52.2) vs. baseline (362.8 + 47.1 min)* **•fSleep efficiency(%):** in PMP (91.9 + 7.8) vs. baseline (85.3 + 7.7)** **WASO (min):** in PMP (39.7 + 36.6) vs. baseline (63.2 + 34.1)** **•fStage N3 (%)**: in PMP vs. baseline. Mean presented in a graphic** **In normal sleep parameters at baseline subgroup (n= 8)** **•fSleep latency (min):** in PMP (6.2 + 5.7) vs. baseline (15 + 6.9)* **In pathological sleep parameters at baseline subgroup (n= 9)** **•fSleep maintenance index (%):** in PMP (92.6 + 4.7) vs. baseline (84.4+7.5)**
	**Hindmarch et al. (2005)^76^**	Randomized, double-blind, placebo and active-controlled, 3-way crossover (class I)	24	Healthy adults	Acute monotheraphy	450mg	Placebo and alprazolam	**Means of nights 2, 3, 4**: **SL (min):** in PGB (6.83±5.55) vs. placebo (13.95+10.78) **•fSE (%):** in PGB (96.11+2.13) vs. placebo (90.62+5.07) **•fTST (min):** in PGB (461.73+10.22) vs. placebo (435.55+24.39) **No. of awakenings (<1min):** in PGB (7.88+4.43) vs. placebo (17.45+6.38) **No of awakenings (>1min):** in PGB (1.30+1.50) vs. placebo (3.84+3.34) **•fSWS (%) in 1st third:** in PGB (66.63+10.35) vs. placebo (46.65+12.97) **•fSWS(%) in 2nd third:** in PGB (31.11+14.89) vs. placebo (19.64+10.04) **•fSWS(%) in 3rd third:** in PGB (16.92+10.8) vs. placebo (10.8+8.61)
	**Haas et al. (2007)^5^**	Randomised, double-blind, placebo- controlled (class II)	15 (PGB n=8)	Focal epilepsy + subjective sleep disturbance	Adjunctive	300mg	Placebo (n=7)	**After 4 weeks, in-home PSG:** **4/No. of awakenings:** in PGB (2.8+5.1) vs. placebo (5.4+3.5)* **•fSleep efficiency (%):** in PGB (87.1+8.9) vs. baseline (80.6+13.6)** **WASO (min):** in PGB (15.1+29.5) vs. baseline (50.5+41.6)* **No. of awakenings:** in PGB (2.8+5.1) vs. baseline (6.0+4.6)*
	**Romigi et al. (2009)^23^**	Prospective, single-blind, uncontrolled (class III)	12	Focal epilepsy	Adjunctive	Individualized (150-375mg/day)	NA	**After 3-months of PGB**: **Stage 2 (%):** in PGB (54.08 + 12.21) vs. baseline (60.44+7.34) **•fREM(%):** in PGB (16.57+7.43) vs. baseline (10.58+6.44)
**Pregablin**	**Garcia- Borreguero et al. (2010)^79^**	Randomized, double-blind, placebo- controlled (class I)	58	Idiopathic RLS	Acute monotheraphy	Mean dose: 337mg/day	Placebo (n=28)	**PGB (n=30) on week 12**: **•fSE:** in PGB (85.67+15.42) vs. baseline (77.71+16.67) vs. placebo (75.76±19.88)** * **WASO:** in PGB (27.18+24.16) vs. baseline (54.71+36.32) vs. placebo (73.46+63.17)** **•fStage N3:** in PGB (60.62+55.16) vs. baseline (46.29+35.83) vs. placebo (35.23+27.01)* **•fStage N4:** in PGB (56.04+49.34) vs. baseline (38.79+35.72) vs. placebo (39.78+35.37)** *
	**Bazil et al. (2012)^80^**	Prospective, randomised, double-blind, crossover (class II)	9	Focal epilepsy + insomnia	Adjunctive	300mg	Placebo	**SWS** increased with PGB 4.1+2.5% vs. Baseline, compared with a drop with placebo of 2.0+1.8% vs. baseline* \p **Stage N1:** check figure in reference for details.*
	**Roth et al. (2012)^78^**	Randomized, double-blind, placebo- controlled, 2- treatment, 2- period, crossover (class I)	102	Fibromyalgia + sleep disturbance	Acute monotherapy	300-450mg/day	Placebo	**After 8 weeks PGB:** **•fTST (min):** in PGB (396.2+4.7) vs. placebo (370.6+4.7)**WASO (min): in PGB (51.5+3.8) vs. placebo (70.7+3.8)** *Sleep latency (min): in PGB (34.5+3.7) vs. placebo (41.6+3.7)* **•fSE (%):** in PGB (82.6+0.98) vs. placebo (77.2+0.97)** * **•fSWS (%):** in PGB (17.2+1.0) vs. placebo (15.0+1.0)** *
	**Garcia- Borreguero et al. (2013)^77^**	Randomizes, double-blind, 3-way crossover (class II)	75	RLS	Acute monotherapy	300mg	Placebo and pramipexole	**After 4 weeks of PGB**: **WASO (min):** in PGB (51.5+4.2) vs. placebo (78.6+4.2)** * **PLMAI, PLMA/hour:** in PGB (3.9+0.7) vs. placebo (7.6+0.7)** *
**Tiagabine**	**Mathias et al. (2001)^81^**	Double-blind, prospective (class I)	10	Healthy adults	Monotherapy	5mg	Placebo	**•fSWS(%):** in TGB (62.9±33.5) vs. placebo (32.8±20.0)* **^Sleep efficiency (%):** in TGB (82.2±4.2) vs. placebo (78.1±5.7)*
	**Walsh et al. (2005)^83^**	Randomized, double-blind, Latin-square design (class I)	22	Healthy elderly	Monotherapy	2, 4 and 8mg	Placebo	**2mg:** no statistically significative differences were found vs placebo **4mg:** **•fTST (min):** in TGB (407.7±33.4) vs. placebo (396.0±31.0)* **WASO (min):** in TGB (64.9+31.8) vs. placebo (77.2+31.9)* **•fSWS (min):** in TGB (59.7±39.1) vs. placebo (44.5±36.8)* **8mg:** **Stage N1 (min):** in TGB (62.4±31.6) vs. placebo (81.3±34.6)** * **•fSWS (min):** in TGB (87.0±61.0) vs. placebo (44.5±36.8)** * **REM (min):** in TGB (58.4±18.1) vs. placebo (68.3±16.6)** **SWS latency (min):** in TGB (33.1±25.8) vs. placebo (51.0±35.3)** **REM latency (min):** in TGB (94.2±48.2) vs. placebo (71.8±30.2)**
	**Walsh et al. (2006)^28^**	Double-blind, randomized, parallel-group, dose-response study (class I)	230	Primary insomnia	Monotherapy	4, 6, 8 and 10mg	Placebo (n=47)	**4mg (n=46):** **4/Stage N1 (%):** in TGB (33.6±19.0) vs. baseline (39.3±22.5)** **6mg (n=45):** **4/Stage N1 (%):** in TGB (25.6±16.8) vs. baseline (32.7±18.0)** * **8mg (n=45):**
								**Stage N1 (%):** in TGB (27.2+18.2) vs. baseline (32.7+18.0)** * **10mg (n=47):** Significance is in change from baseline vs. placebo.
	**Walsh et al. (2006)^84^**	Double-blind, randomized, five- period, Latin- square, crossover (class I)	50	Primary insomnia	Monotherapy	4, 8, 12 and 16mg	Placebo	**4 mg (n=50):** **•fSWS(%):** in TGB (10.2+1.3) vs. placebo (5.7+1.3)*REM(%): in TGB (18.5+0.6) vs. placebo (20.2+0.6)*8mg (n=50): **Stage N1 (%):** in TGB (8.2+0.6) vs. placebo (11.0+0.6)** **•fSWS(%):** in TGB (13.1+1.3) vs. placebo (5.7+1.3)*REM(%): in TGB (16.8+0.6) vs. placebo (20.2+0.6)** *12mg (n=50): **Stage N1 (%):** in TGB (7.3+0.6) vs. placebo (11.0+0.6)** * **Stage N2 (%):** in TGB (58.9+1.1) vs. placebo (63.2+1.1)** **•fSWS(%):** in TGB (20.0+1.3) vs. placebo (5.7+1.3)** *REM(%): in TGB (13.8+0.6) vs. placebo (20.2+0.6)** *16 mg (n=50): **WASO (min):** in TGB (55.9+3.9) vs. placebo (66.9+3.9)*Stage N1 (%): in TGB (7.8+0.6) vs. placebo (11.0+0.6)*Stage N2 (%): in TGB (53.6+1.1) vs. placebo (63.2+1.1)** * **•fSWS(%):** in TGB (27.5+1.3) vs. placebo (5.7+1.3)** * **REM(%):** in TGB (11.1+0.6) vs. placebo (20.2+0.6)*
	**Walsh et al. (2006)^82^**	Radomized, double-blind, parallel-groups (class I)	38	Healthy volunteers	Monotherapy	8mg	Placebo	**Night 2 versus mean of nights 3-6** **SWS:** More [29.1 (± 23.8)] minutes of SWS on nights 3 to 6 compared to baseline vs. placebo [5.4 (± 22.1)] fewer minutes compared to baseline.
	**Roth et al. (2006)^85^**	Radomized, double-blind, parallel-groups (class I)	207	Elderly with primary insomnia	Monotherapy	2, 4, 6, 8mg	Placebo (n=38)	**2mg (n=42):** no significative changes from baseline vs. placebo **4mg (n=38):** **Stage N1 (min):** in TGB (31.2+2.8) vs. placebo (39.1+3.1)* **SWS(min):** in TGB (44.2+5.6) vs. placebo (35.2+4.4)* **6mg (n=45):** **Stage N1 (min):** in TGB (29.7+2.6) vs. placebo (39.1+3.1)*SWS(min): in TGB (72.2+7.9) vs. placebo (35.2+4.4)*REM (min): in TGB (50.6+3.0) vs. placebo (63.7+2.9)* **No. 30s-awakenings:** in TGB (30.6+1.3) vs. placebo (35.4+1.8)* **•fRatio SWS/(Stage N1+WASO) (%)::** in TGB (0.9+0.21) vs. placebo (0.3+0.005)*
								**8mg (n=41):** **Stage N1 (min):** in TGB (30.5+3.2) vs. placebo (39.1+3.1)*SWS(min): in TGB (70.0+8.3) vs. placebo (35.2+4.4)*REM (min): in TGB (44.5+3.8) vs. placebo (63.7+2.9)* **No. 30s-awakenings:** in TGB (32.2+1.8) vs. placebo (35.4+1.8)* **•fRatio SWS/(Stage 1+WASO) (%)::** in TGB (0.6+0.1) vs. placebo (0.3+0.005)*
	**Feld et al. (2013)^86^**	Randomised, double-blind, crossover (class I)	12	Healthy adults (only men)	Monotherapy	10mg	Placebo	**Stage N1 (%):** in TGB (4.23+1.16) vs. placebo (8.44+1.08)** **•fSWS (%):** in TGB (24.30+3.34) vs. placebo (17.51+0.89)** **REM(%):** in TGB (12.37+2.14) vs. placebo (17.51+0.89)*
	**Taranto- Montemurro et al. (2017)^87^**	placebo- controlled, double-blind, crossover trial (class I)	12	Obstructive apnea	Monotherapy	12mg	Placebo	No significative differences were found vs. placebo.
**Topiramate**	**Bonnani et al. (2004)^88^**	Prospective, non- randomized, non- blind (class III)	14	Focal epilepsy	Monotherapy	200mg/day	14 healthy controls	**After 2 months of TPM:** Sleep parameters in PSG reported no significative changes. MSLT scores did not significantly change in patients as compared with pretreatment values.
**Zonisamide**	**Romigi et al. (2013)^90^**	Open-label prospective (class III)	Baseline (n=12) Follow-up 3 months (n=12)	Focal epilepsy	Adjunctive	200-300mg/day	NA	**No. Of awakenings** in ZNS (12.72+10.65) vs. baseline (9.55±5.65)*
	**Eskandari et al. (2014)^89^**	Randomised, double-blind, parallel study with open extension phase (class II)	47 (ZSM, n=22)	OSA	Adjunctive	300mg	NA	**Sleep-related variables at 4 weeks:** no significative changes.

Abbreviations: FEL = Felbamate; GBP = Gabapentin; LAC = Lacosamide; LTG = Lamotrigine; LEV = Levtircetam; PMP = Perampanel; PGB = Pregabalin; TGB = Tiagabine; TPM = Topiramate; ZNS = Zonisamide.

* *p*<0,05;

** *p*>0,01;

*** *p*<0,001.

**Table 3:** Summary of studies on more than one antiepileptic drug (n=7).

Authors	Type of study	N	Population	AED studied	Type of therapy	Dose (per day)	Comparison	Significative PSG results (mean ± SD)
**Wolf et al (1984)^51^**	Prospective, non-blind, non- randomised (class III)	Focal epilepsy (n=15) PB (n=34)	PHB monotherapy. 4 cycles and crossover	Phenobarbitone (n=34) and phenytoin (n=34)	Placebo	Daily dose. Steady state serum level PB=26.8pg/ml (10-46)	Phenobarbitone (n=34) and phenytoin (n=34)	1st Cycle: **•fREM (min):** in PB (126.4+44.9) vs. baseline (95.3±39.7)* **•fREM latency (min):** in PB (112.9+40.6) vs. baseline (81.7+33.8)* **•fStage N4 (min):** in PB (45.1+30.1) vs. baseline (25.4+13.5)*
								2nd Cycle: **Time awake (min):** in PB (1.7+2.0) vs. baseline (4.6+8.6)* **•fREM (min):** in PB (117.1+21.9) vs. baseline (108.1+25.6)** **•fREM latency (min):** in PB (220.8+50.2) vs. baseline (175.5+50.6)** **•fStage N2 (min):** in PB (58.5+25.3) vs. baseline (38.7+15.7)* **•fStage N3 (min):** in PB (32.0+19.1) vs. baseline (22.5+14.4)** **•fStage N4 (min):** in PB (13.3+7.4) vs. baseline (21.2+16.6)**
								3rd Cycle: **Time awake (min):** in PB (1.8+2.6) vs. baseline (3.8+3.0)** **•FStage N4 (min):** in PB (3.5+7.4) vs. baseline (8.0+10.3)** **•fREM latency (min):** in PB (281.8+48.2) vs. baseline (321.9+52.1)** **•FStage N1 (min):** in PHE (4.0+3.9) vs. baseline (8.7+6.8)* **•FStage N2 (min):** in PHE (41.9+17.3) vs. baseline (54.9+17.4)** **•fStage N3 (min):** in PHE (14.4+10.3) vs. baseline (9.9+10.6)** **•fStage N4 (min):** in PHE (17.7+15.3) vs. baseline (8.0+10.3)*
								4th Cycle: **Time awake (min):** in PB (1.3+0.8) vs. baseline (3.2+4.0)* **•fREM latency (min):** in PB (403.1+50.7) vs. baseline (366.1+47.9)** **•fStage N2 (min):** in PB (51.5+17.3) vs. baseline (41.8+14.1)* **•fStage N3 (min):** in PB (36.9+18.3) vs. baseline (26.4+12.8)** **•fStage N4 (min):** in PB (1.6+3.6) vs. baseline (6.2+10.6)** **•fStage N3 (min):** in PHE (11.5+8.3) vs. baseline (7.8+10.1)*
**Wolf et al. (1984)^47^**	Observational, nonrandomized (class III)	229	Absence seizures	ETH (n=143) VPA (n=187)	Monotherapy	NA	NA	**No values available:** **•fStage N1 (%):** in ETH vs. baseline* **SWS (%):** in ETH vs. baseline* **•fREM duration (min):** in ETH vs. baseline in third REM cycle * **>V REM duration (min):** in ETH vs. baseline in fourth REM cycle * **•fStage N1 (%):** in VPA vs. baseline*
**Drake et al. (1990)^44^**	Observational (class III)	17	Focal or generalized epilepsy	PHE (n=5), CBZ (n=5), VPA (n=5), CNZ (n=2)	Chronic monotherapy	NA	NA	PHE (n=5), CBZ (n=5), VPA (n=5), CNZ (n=2) **SWS and REM:** in both groups compared to published norms but none values were presented.
**Manni et al. (1993)^52^**	Observational (Class III)	30	Generalized epilepsy treated with PB; Focal epilepsy treated with VPA.	PB (n=10) VPA (n=10)	Chronic monotherapy	PB (mean serum = 19.3±1.7pg/ml) VPA (mean serum = 85.7±4.7g/ml)	Healthy controls (n=10)	MSLT: **Daytime sleep propensity:** in PB vs. controls and vs. CBZ **No significative differences in PSG recordings among the 3 groups.**
**Bonanni et al. (1997)^40^**	Prospective, non- randomized (Class III)	26	Focal epilepsy	CBZ = 26 CBZ+VGB= 14	Chronic CBZ monotherapy (n=26) CBZ + VGB (n=14)	CBZ: 600-1,600mg VBB: 2-3g/day	Healthy controls (n=12)	No significative differences were found.
**Legros et al. (2003)^38^**	Observational, uncontrolled (class III)	39	Focal epilepsy with at least 24-h seizure-free		Monotherapy	Each drug with different dose	Patients with no AED as controls (n=13)	**PHE (n=7):** **•fStage N1 (%):** in PHE (13.2+7.3) v. controls (7.7±4.8)**; **SWS (%):** in PHE (7.9±4.2) v. controls (11.3+4.4)*; **REM (%):** in PHE (13.9+6.2) v. controls (18.8+5.1)*; **VPA (n=2):** **•fStage N1 (%):** in VPA (16.8+9.8) v. controls (7.7±4.8)**;
								No significative differences were found in **CBZ (n=10), PB (n=1), GBP (n=3), LTG (n=4),** **PRI (n=1), FBM (n=1), ZNS (n=1), TGB (n=1).**
**Cho et al. (2011)^37^**	Longitudinal randomized trial (class II)	31	Newly diagnosed focal epilepsy	LEV (n=16) CBZ-CR (n=15)	Monotherapy	LEV (1,000mg/day) CBZ-CR (400mg/day)	NA	**LEV group (n=16)** **Sleep efficiency (min):** on LEV (90.62±4.77) vs. baseline (84.32±13.65)* **WASO (min):** on LEV (31.72±38.88) vs. baseline (55.61±56.06)* **CBZ-CR group (n=15)** **“TStage N3 (%):** on CBZ-CR (26.78±9.47) vs. baseline (21.29±9.76)*

Abbreviations: PB = Phenobarbitone; PHE = Phenytoin; ETH = Ethosuximide; VPA = Valproic acid; CNZ = Clonazepam; CBZ = Carbamazepine; GBP = Gabapentin; LTG = Lamotrigine; FBM = Felbamate; ZNS = Zonisamide; TGB = Tiagabin; CBZ-CR = Carbamazepine-controlled release.

* *p*<0,05;

** *p*>0,01;

*** *p*<0,001.

We found twenty-one randomized, double-blind studies, placebo-controlled (class I evidence), nine class II studies, thirty-one class III and two class IV considering the effects of AEDs with objective measure of sleep architecture parameters.

### Classic antiepileptic drugs

#### Carbamazepine

Seven studies were obtained concerning carbamazepine (CBZ), with the best evidence being a class II trial in borderline personality disorder^31^ and the rest were all class III of evidence. This AED was primarily associated with a significative increase in SWS and a concomitant decrease in the REM stage in a class III study performed in healthy volunteers^32^. Subsequent trials confirmed this by using CBZ in acute treatment of patients with focal epilepsy^33,34^, in psychiatric disorders^31^, and in healthy volunteers^35^. In a smaller study, conducted in healthy individuals, CBZ also appeared to improve sleep continuity and efficiency^36^. Another longitudinal trial reported an increase in SWS after using CBZ in PWE, but without significant differences concerning the REM stage^37^. Gann et al. (1994)^35^ described a higher SE and a decrease in N2 stage.

The impact of chronic CBZ treatment in sleep parameters was assessed by Manni et al. (1990)^33^ on one observational, small sample, non-controlled study in focal epilepsy subjects. These authors found an overall decrease in the latency and percentage of REM stage, a reduction in sleep stability, with a greater number of stage shifts, and a reduction in the number of awakenings. However, it is necessary to consider that unstable sleep patterns and REM decrease more frequent in the subgroup of patients with poor seizure control so that sleep unstability seems to be related more to seizures than to CBZ in itself^33^. In the opposite, Legros et al. (2003)^38^ reported a non-significant decrease in REM sleep and supported the idea that chronically administered CBZ does not significantly affect sleep of focal epilepsy patients.

In most recent articles, some sleep parameters, such as higher cyclic alternating pattern rates during NREM and arousals, appear to be worse than in drug-naïve individuals. Therefore, the authors concluded that CBZ negatively affects sleep quality in this subgroup of epileptic patients. Since drug exposure was not controlled, it is not possible to conclude whether sleep changes are caused by the underlying disease or by exposure to CBZ^39^.

Although CBZ initially has a deleterious effect on sleep, namely through the reduction of REM sleep, in the long-term, it does not seem to significantly modify this parameter. The authors suggest that those effects may only be acute and temporary^34,38,40^.

Despite of CBZ being extensively used in neuropathic pain such as trigeminal neuralgia, we have not found trials concerning these patients’ group.

The low level of evidence, due to the lack of class I studies, prevents definitive conclusions about the impact of CBZ on sleep structure. However, in acute therapy, most articles seem to point to an increase in SWS and a decrease in the REM stage. In chronic intake, there is less evidence of sleep disturbances but the majority of the evidence point towards an innocent profile.

#### Clobazam

Most clinical trials studying the influence of clobazam (CLB) on sleep were done in children and, therefore, these articles could not be included in this review.

Being mainly used as a sedative or a hypnotic drug it is a surprise that the evidence available in adults comes out from only one class I, randomized, double-blind study in healthy volunteers performed by Nicholson et al. (1977)^41^; the authors compared doses of 10mg and 20mg of CLB. In both doses, this benzodiazepine drug reduces SL and improves SE but the lower dose decreased the percentage of stage N1 and SL compared to placebo. With the higher dose, the effects on stage N1 and SL were more pronounced, with the authors also describing a decrease in stage N3 and SWS and noting an increase in stage N2. No changes in REM sleep was noted in this study^41^.

#### Clonazepam

Although clonazepam (CLN) is extensively used in sleep disorders, the available literature on its impact on sleep structure is scarce^42^. Only five articles were found, four of which class III and only Sakai et al. (2017)^43^ present a class I study. They reported nonsignificant changes in patients with sleep bruxism undergoing CNZ compared to placebo. This remains the highest quality evidence on CLN although we can consider that the crossover with clonidine can be a confounding factor for the results presented.

Drake et al. (1990)^44^ were the first authors to study CLN’s influence on sleep. This trial was conducted in healthy adult volunteers and they found a reduction in SL and REM latency. Saletu et al. (2010)^45^ described a relatively positive sleep profile with 1mg of CLN compared to placebo in patients with sleep bruxism^45^; these patients suffered from fewer awakenings, longer TST, reduced WASO, greater SE, less stage shifts and an increase in stage N3, while stage N1 decreased.

In an observational study conducted in patients with idiopathic REM sleep behavior disorder (iREMSBD), the authors described the lack of objective effects of CNZ in REM sleep parameters, but evidence of NREM sleep disturbance was found in a group of these patients on chronic use of CNZ. The retrospective design, the heterogeneity of sample size and disease’s duration of the study decreases its level of evidence. Nonetheless, after 2,5 years of follow-up, they concluded that CLN decreases NREM sleep instability, increases N2 stage and decreases WASO relative to baseline^42^. The author confirmed these findings with a new longitudinal study also in iREMSDB patients, but no significant results were found in those undergoing acute monotherapy^46^.

#### Ethosuximide

The only article found on ethosuximide was an observational class III study conducted in PWE, with absence seizures, by Wolf et al. (1984)^47^; they found a significant increase in N1 sleep and a concomitant reduction in SWS.

#### Phenobarbital

Phenobarbital (PB) is a sedative agent, sometimes used as an hypnotic drug. For this reason, evident changes in sleep architecture were expected^48^. Four articles on PB were found, of which only one was class I and the others were only class III of evidence.

The best evidence comes from a study performed by Karacan et al. (1981)^49^ in healthy individuals that found a dosedependent reduction in REM sleep, the main change of PB in sleep architecture. The authors also reported a reduction in stage shifts and number of awakenings and an increase in SL and N2 stage. Also, in healthy individuals, Prinz et al. (1981)^50^ found no differences in immediate acute monotherapy compared to placebo. However, after one month taking PB, a significant reduction in SL and SWS was observed^50^.

On PWE, Wolf et al. (1984)^51^ found remarkable acute changes in PSG compared to baseline. In generalized epilepsy, PB diminished REM interruptions and showed a tendency to decrease SWS. On MSLT, Manni et al. (1990)^52^ reported a greater propensity to daytime sleep in generalized epilepsy treated with PB compared to focal epilepsy treated with CBZ or healthy controls. However, no changes in sleep architecture were found in PSG sleep variables.

In summary, the most evident impact of PB in sleep architecture is a dose-dependent reduction in REM sleep. It appears to also reduce N3 stage in chronic, after a month, intake. There is also an increase in a latency to sleep either in patients with epilepsy or healthy adults.

#### Phenytoin

This review included four articles concerning phenytoin (PHT) use in PWE. Unfortunately, all of them were class III evidence.

Roder-Wanner et al. (1987)^53^ conducted the only trial in which PHT is studied alone. They conclude that PHT increased SWS, while reducing NREM sleep, and managed to differentiate a modest acute change in sleep, from a more prominent 6-weeks effect; these authors also stated that, with continuous administration, the third REM-cycle was consistently the most affected and found a diminished sleep latency.

In a crossover study with phenobarbital, Wolf et al. (1984)^51^ also reported an increase in SWS and decreased sleep latency, but without a significant reduction in REM stage. In an observational study, Drake et al. (1990)^44^ included five patients chronically treated with PHT and noticed a decreased in TST. Like Wolf et al. (1984)^51^, these authors found little to no effect on REM sleep, but, conversely, they noticed an increase in sleep latency. Despite the discordant results, we need to consider that this study was conducted in a very small sample and with uncontrolled PHT dose-exposure.

Legros et al. (2003)^38^, in an observational and uncontrolled trial, found an increase in N1 stage, while observed a modest decrease in the REM stage, in agreement with previous studies. However, they reported a decrease in SWS, conflicting to previous studies. The small sample of this trial, including only seven PWE, limits the conclusions’ validation. Additionally, it also has the disadvantage of including patients with an acute withdrawal of AEDs, whose effects on sleep cannot be excluded.

All studies obtained on PHT were class III. This prevents the establishment of solid conclusions about the drug. However, from what we have obtained we can infer a possible impact in every stage reducing N1, N2 and REM while increasing SWS. Conflicting results were found in terms of sleep latency.

#### Valproic acid

Five articles regarding valproic acid (VPA) were eligible for this review but only one was class II and the rest was class III of evidence.

The best evidence comes from a double-blind, placebo controlled crossover with levodopa trial in patients with RLS in which VPA appears to have an innocent impact in sleep parameters, except for increased N2 latency compared to placebo^54^. In most studies, VPA has been associated with attention impairment during the day by increasing N1 sleep and decreasing SWS^44,55^, however minimal to no effects on sleep architecture were demonstrated. Weight gain is a well-known adverse effect of VPA which itself can lead to sleep disruption, so more studies considering this variable are needed^44,54,56^.

In healthy adults, 500mg per day of VPA appears to reduce REM stage and increase SWS, these changes are more pronounced with 1,000mg/day compared to placebo^56^.

In PWE, both Wolf et al. (1984)^47^ and Legros et al. (2003)^38^ reported an increased N1 stage as the only change in the sleep parameters of the VPA subgroup. Drake et al. (1990)^44^ reported a slight increase in SWS, but their observational study only included five patients on VPA monotherapy and they used an ambulatory EEG record instead of an in-lab PSG. Nayak et al. (2016)^57^ conducted the first VPA study in juvenile myoclonic epilepsy, reporting a slight increase in REM sleep macrostructure in patients under VPA compared to drug naïve patients. This study focused mainly on sleep microstructure.

### Newer antiepileptic drugs

#### Felbamate

The only published study on felbamate (FBM) was a case report in a PWE that suggested a reduction in phase-shifts, nocturnal awakenings, N1 stage and WASO and an increase in the sleep efficiency index^58^. This class IV evidence is insufficient to draw conclusions about FBM effects on sleep.

It is known that FBM has a stimulating profile, being responsible for insomnia, anorexia and anxiety in focal epilepsy, lacking anxiolytic effects^58^. Thus, a formal evaluation of sleep parameters should be performed.

#### Gabapentin

Gabapentin (GBP) is one of the best studied AEDs, with nine articles included in our review, two of them class I, three class II, three class III and one class IV of evidence. It is curious that, although GBP is widely used in neuropathic pain, no study has been designed in this pathology. The most common findings, reported in almost every study about GBP, is an increase in SWS and reduction of N1 stage, with variable effects in REM sleep^59^.

The most objective and robust evidence on GBP was provided by a large multicenter, randomized class I clinical trial. The authors studied the GBP effect on sleep compared to placebo, in adults without epilepsy, but with sleep complaints. A dose of 250mg and 500mg of GBP was administered thirty minutes before bedtime and the results demonstrated an increase in TST and in sleep depth in both gabapentin users. Therefore, GBP has beneficial effects on sleep, contributing to a longer sleep duration and greater depth of sleep^60^. Another class I trial also in healthy adults confirmed an increase in TST and a decrease in WASO, in line with the previous studies. Authors also found a small but significant decrease in the REM percentage and slight increase in the N1 stage^61^.

The oldest GBP study was a class II trial conducted in six healthy adults, so it is not possible to extrapolate the results. The authors found that GBP had no effect on TST, REM or REM latency, but increased SWS and decreased sleep latency^62^. In another class II sleep bruxism patients’ trial, GBP contributed to a significant reduction of bruxism episodes per night, suggesting that it could be an effective alternative treatment. This AED significantly increased the TST, SE and N3 stage compared to a stabilization splint^16^.

Although GBP worsens apnea and respiratory indexes in healthy non-obese adults, the most recent class II trial on sleep apnea found no significant effects on sleep architecture^63^. Legros et al. (2003)^38^ conducted a class III observational study that, among other AEDs, contained a subgroup with only three PWE using GBP, they found no significant differences between these drug-users versus placebo. Due to its small sample, it is not possible to generalize these results.

An open-label class IV study with GBP reported an increase in SE and SWS and a decrease in the arousal index, without affecting any other sleep parameters. The methodological flaws of this study were highlighted in a letter to the editor, published in the same journal, questioning its real relevance to the current evidence^64,65^.

#### Lacosamide

Two studies concerning lacosamide (LCS) and sleep architecture were obtained. Foldvary-Schaefer et al. (2017)^66^ perform the first class I trial in PWE that provide the most robust evidence, that indicated that LCS is not a major contributor to EDS, although there is an increase in prevalence of EDS in these patients. At baseline and at follow-up, the only significant change compared to placebo was a reduction in the arousal index, suggesting a positive effect of LCS use. However, the study’s short duration and the recruitment from a single site limit the generalization of these findings. This innocent role of LCS in sleep is supported by previously published class III research in healthy individuals^67^.

#### Lamotrigine

Three articles focused on the use of lamotrigine (LTG) in focal epilepsy patients were obtained. All of them are class III of evidence. Clinically, LTG is not associated with EDS and most studies indicate an improvement in sleep stability^20,68^. Legros et al. (2003)^38^ found no statistically significant differences in sleep architecture in four epileptic patients using LTG. The other two studies, both open label, found a reduction in SWS. Placidi et al. (2000)^20^ found a significant increase in REM sleep, a reduction in the number of REM entries and phase shifts and a lack of negative effects on daytime somnolence in drugresistant PWE using LTG as a chronic add-on medication. On the other hand, in focal epilepsy subjects, Foldvary et al. (2001)^68^ found an increase in the N2 stage and less stage shifts and arousals, suggesting LTG as a less sleep-disturbing drug than classic AEDs.

There is still no evidence regarding the effects of LTG on healthy individuals to allow conclusions, but in PWE it is associated with positive effects on sleep. We have also found no studies on mood disorders, even though LTG is widely used as a mood stabilizer.

#### Levetiracetam

Levetiracetam (LEV) has been recently associated to a higher total sleep duration, even though, sleep complaints are not a commonly reported adverse effect of this drug^69^. We found six reports evaluating sleep parameters in subjects taking LEV with very heterogeneous results. Two were class I, two were class II, and two were class III of evidence.

Bell et al. (2002)^70^ designed two studies; the first was a class I trial conducted in healthy individuals and the second one was a class II trial performed in focal epilepsy patients taking concomitantly CBZ. They concluded that LEV increased the N2 stage, in both groups. In healthy adults, LEV increased REM latency and decreased SWS sleep in patients with focal epilepsy. Unlike BZD, it does not seem to affect sleep continuity, although a subjective reduction in sleep perception was observed^70^. The best evidence on LEV that we found came from a study performed by Cicolin et al. (2006)^71^. It is a randomized, doubleblind design with higher dose of LEV 2,000mg/day, in healthy volunteers, that found and reinforce the increase in the N2 stage. In MSLT the sleep latencies were normal and they also did not find any abnormalities in MSLT and thus the authors considered that LEV at therapeutic dose for epilepsy stabilizes sleep without affecting daily activities.

In a small class II study performed by Bazil et al. (2005)^18^, LEV seemed to have only minor implications in healthy volunteers sleep structure, with the only relevant difference being an increase in the number of awakenings. This was in accordance with the findings described by Zhou et al. (2012)^27^ that only observed a reduction in REM sleep time after LEV treatment with a dose of 1,000mg/day, on PSG evaluation; they reported no changes in MSLT.

Using actigraphy and MWT on focal epilepsy subjects, Yilmaz et al. (2007)^72^ observed that, after three weeks of LEV, there was an increase in daytime napping episodes and total nap duration while there was a decrease in total activity score at night as in monotherapy as in an add-on regimen. The authors point out that although actigraphy gives a good measure of sleep continuity, it is not as efficient as PSG in assessing sleep architecture and these results must be confirmed.

In a crossover trial with CBZ in newly diagnosed focal epilepsy patients, Cho et al. (2011)^37^ described that LEV increased sleep efficiency, reduced WASO and did not have overall major effects in sleep structure as opposed to those on CBZ.

#### Perampanel

We obtained only three articles regarding the effects of perampanel (PER) on sleep parameters^17,73^. All were class III of evidence, with uncontrolled design and with a small sample, two of them were done in PWE and the other one was performed in patients with restless legs syndrome (RLS).

PER is known to cause EDS. This is the most commonly reported adverse effect in these trials and, therefore, it is recommended to be taken before bedtime^17^. A positive sleep profile was subjectively described when PER was used as an adjunctive AED. In an exploratory study done by Gonzalez-Cuevas et al. (2017)^73^, in patients with focal epilepsy in adjunctive treatment with PER, this AED was well tolerated and did not cause any somnolence or modification in sleep parameters. Garcia-Borreguero et al. (2017)^74^ comment on these findings in their open-trial in RLS patients. Unlike previous authors, they described many modifications on objective sleep outcomes. In monotherapy, PER increase TST, SE and arousals, and decreased SL and WASO. Regarding the sleep stages, the SWS increased and the N1 stage decreased. It did not affect the duration or percentage of REM, but it did reduce its latency. Rocamora et al. (2020)^75^ in the most recent pilot study corroborated this positive profile of PER but this time in patients with epilepsy. They also reported a decrease in sleep latency in the subgroup of patients with normal sleep parameters on baseline.

There are still no large randomized, controlled clinical trials on PER to establish definitive conclusions.

#### Pregabalin

Seven articles were obtained on the effect of pregabalin (PGB) on sleep parameters. Three of which were class I evidence and three class II and one class III. PGB is also used in pain but no article comprising this subgroup of patients was selected for review.

The only randomized clinical trial conducted in healthy adults described an increase in TST and in SE and a decrease in SL and in the number of awakenings, shorter or longer than one minute. Regarding sleep stages, the SWS increased, and the duration of REM sleep was reduced. No disturbances were found in REM latency^76^. Another randomized study, conducted in patients with RLS, had the most controlled design conducted on a total of seventy-five PGB users. The authors found a reduction in WASO and the number of awakenings, making it better than placebo in improving sleep disturbances, as assessed by objective and subjective sleep parameters^77^. A class I study in adults with fibromyalgia and sleep complaints found similar results with an overall improvement of sleep^78^.

Garcia-Borreguero et al. (2010)^79^ found an increase in SE, an increase in the percentages of stages N1, N2 and N3 and in minutes of SWS and WASO, after three weeks of PGB in patients with idiopathic RLS. There were no changes in REM, in TST, or sleep latencies.

In patients with focal epilepsy, after four weeks of adjuvant therapy with PGB in individuals with sleep complaints, an improvement in sleep continuity was demonstrated in a class II study. This was due to a reduction in WASO and in the number of awakenings and an increase in SE. The authors complain about the small sample used, mentioning difficulties in recruiting patients^5^. In a similar group of patients, Bazil et al. (2012)^80^ did not find any of these changes but in turn found a significative increase in SWS percentage and a modest reduction in stage N1 sleep. In a group of twelve patients with focal epilepsy without sleep complaints, after three months adjuvant therapy with PGB, Romigi et al. (2009)^23^ reported only a significant increase in REM sleep and a reduction in the N2 stage compared to baseline.

#### Tiagabine

Tiagabine (TGB) is the best studied AED regarding objective sleep parameters, with nine articles obtained, seven of them are class I. The other two trials were class II and class III of evidence.

Mathias et al. (2001)^81^ were the first to describe a higher number and frequency of arousals in placebo group, rather than in the TGB group, in healthy subjects at a 5mg daily dose. The authors showed that patients from TGB group have greater sleep efficiency and SWS.

During a sleep restriction period of four nights and with 8mg of TGB, Walsh et al. (2006)^82^ were the first authors to describe an improvement in the SWS compared to baseline and placebo. In an exploratory study with healthy elderly individuals, Walsh et al.^82^ found a positive effect on sleep with a 4mg or 8mg daily dose of TGB. No differences were found between placebo and 2mg daily dose of TGB. Those results demonstrated an improved sleep maintenance by decreasing WASO and/or stage N1 as well as increasing SWS^83^. On this study, patients had no subjective complaints concerning sleep and the reduction found in stage N1 may result from increasing time passed on SWS, which reduces arousability. Only studies with a larger sample will provide a clear assessment of the various doses of TGB in the PSG variables^83^.

In older adults with primary insomnia, TGB shows a dose-dependent increase in SWS^84,85^, at daily doses up to 8mg. It does not appear to increase sleepiness in the following morning, but it did show a reduction in the duration of stage N1^85^.

In a study by Walsh et al. (2006)^82^ TGB seems to decrease the biological consequences of sleep restriction of healthy adults. However, in a subsequent trial, memory consolidation did not improve^86^. A double-blind crossover study by Feld et al. (2013)^86^ showed that TGB improves SWS and decreases REM without affecting subjective sleep perception or improving memory consolidation. They proposed that TGB-induced SWS is functionally different from normal SWS. This may be the possible reason why it fails to improve memory consolidation. WASO, LPS, and TST, which are the traditional variables of hypnotic efficacy, were not modified by TGB in any dose in a study performed by Walsh et al. (2006)^28^. Only the group of patients with a TGB dose of 10mg, the maximum daily dose in the study, reported morning sedation. In the largest sample study, TGB significantly increased SWS at any dose, while decreasing N1 stage^28^. Compared with placebo, TGB showed an increased in slow-wave activity on EEG, but no SWS duration abnormalities during sleep were observed. It has also failed to increase N3 sleep in patients with obstructive sleep apnea^87^.

#### Topiramate

Only one class III article was selected. Bonanni et al. (2004)^88^ reported the absence of significant changes in objective sleep parameters in PSG or MSLT in PWE taking topiramate (TPM). Interestingly in this study is that, it goes against the common subjective description of drowsiness related to TPM. Although prospective, the quality of the evidence provided by this study is low, due to its lack of randomization or concealment. Additional studies are needed, as topiramate (TPM) is an extensively used medication, not only in epilepsy but also in other diseases, such as headaches, essential tremor, impulsivity behavior control and weight loss^88^.

#### Vigabatrin

Bonanni et al. (2004)^88^ reported no changes in sleep structure when vigabatrin (VGB) was used as an add-on to CBZ in focal epilepsy. However, some cases described a higher subjective level of somnolence compared to CBZ monotherapy^40^. This class III study was not a randomized trial, nor had a blinded intervention, but it remains the only objective sleep study on VGB.

#### Zonisamide

Two articles, one class II and another one class III of evidence were collected. In a class II randomized, placebocontrolled but with an open-label extension phase study on patients with sleep apnea, Eskandari et al. (2014)^89^ reported no changes in sleep parameters within four weeks of treatment. Sleep apnea events were less frequent with zonisamide (ZNS), regardless of the reduction in body weight, probably due to the inhibition of carbonic anhydrase mechanism. This, theoretically, could result in an improvement of EDS, but this benefit was not seen in the study.

In their observational study, Legros et al. (2003)^38^ included one patient with a focal epilepsy treated with ZNS. They found no significant changes in sleep parameters compared to healthy controls. A prospective study made by Romigi et al. (2013)^90^ in focal epilepsy reported only a slightly greater number of awakenings.

#### Other AED

We found no articles concerning brivaracetam, eslicarbazepine acetate, rufinamide or stiripentol effects on objective sleep parameters. Recently Assenza et al. (2018)^91^ in their multicenter study pointed out that ESL led to lower subjective feelings of drowsiness, so its objective study is of great interest.

Studies concerning primidone, sulthiame and oxcarbazepine are reported in previous literature but were done either in pediatric population or in animals and thus could not be included in this review^92,93^.

## DISCUSSION

The study of the effects of AED on sleep architecture is a complex task given the intricacy of several factors. First of all, because both epilepsy and sleep interact. The sleep/wake cycles influence the moment of seizure occurrence and seizure affect the sleep structure (staging, sleep duration and cyclical pattern). This was shown in previous studies, not included in this analytical one because they are not easily accessed on current databases. Studies on book chapters and reviews frequently are not included in meta-analysis, with limitations on field knowledge. However, as was previously described on the literature for sleep-epilepsy-AED interactions^19^ this analytical review highlight diverse reasons as decisive for the inconsistence of the results. This because studies on sleep-epilepsy-AED interactions: i) use different methodologies for classification and structure of sleep - methodologies often not objective; ii) compare results of sleep studies in PWE with different epilepsies, with the results obtained by the use of AED in other pathologies; iii) compare results of sleep structure on PWE with different therapeutic regimes - inducing different AED distribution profiles; iv) compare results of influence of AEDs in the sleep of PWE frequently not taking into account (lately) previous treatments; v) frequently had scarce information on the control of the epilepsy - and consequently on interference of seizures on sleep - namely of focal epilepsy, with little or non-exuberant symptomatology that occur in sleep; vi) frequently interactions with other AEDs or other co-medications are not full known; and vii) because the influence of AED on sleep architecture encompass conclusions from studies in children in which the sleep structure and maturation is not the same as in adults.

In addition to these difficulties most of the studies found in this review with focus on the effect of AED in sleep architecture parameters were non-controlled studies or retrospective series of cases that have class IV of evidence, so in most of the AEDs any conclusion can be suggested, and in many others only a weak evidence-based recommendation can be established.

One of the objectives of this review was to compare classic with newer AEDs in terms of objective sleep parameters. After reviewing the literature and based on the reported results, it seems that newer AEDs are associated with less modifications in sleep architecture.

Among the classic AEDs, CBZ is the best studied AED. Its effects on sleep go through a reduction in REM phase and an increase in SWS. On other hand, PB seems to have an innocent or slightly dose-dependent interference effect on sleep. In most of the studies analyzed, VPA seems to have little to no effect on sleep architecture. PHT studies have low evidence level and conflicting data regarding its effect on sleep. Therefore, in absence of better data, we will not draw conclusions. All ethosuximide evidence found was in a class III article not designed for this drug. In spite of being benzodiazepines CLB and CLN results does not suggest a REM sleep reduction, which is surprising, appearing to have a positive effect in sleep structure. However, as most studies of CLB are designed in children we could have lost important data limiting this review to adults.

In the newer AEDs group, there are some AEDs, such as GBP, LCS and PGB, which seem to have an overall positive profile in sleep structure. In PWE, LTG also appear to have a positive profile, but there is a lack of sufficient evidence to suggest a recommendation. TPM appears to be innocent in sleep architecture and it would be interesting to have new randomized studies, with larger samples to confirm this assumption. Others, such as LEV, despite improving SE and TST and not being associated with clinically evident EDS, caused changes on sleep architecture. These changes were not considered positive, once, it increased stage N2 or reduced REM. There are AEDs in which we cannot draw any conclusions like FBM, because there are no randomized studies yet.

In our review, we only selected objective studies in the qualitative analysis. A limitation of our search was that the articles collected had very heterogeneous methodologies, either in terms of the objective method used (PSG, MLTS, or actigraphy) either in terms of dose of AED studied. Another point is that studies sometimes used old parameters like stage N4, because they predate the new classification, and the validity and transposition of results from the old classification on NREM S3-4^7^ could not be directly to NR3 on the new^9^, current sleep classification^10^. It was difficult to interpret results from very small samples and extrapolating conclusions in this context. As lab PSG is being less used for the diagnosis and characterization of sleep, frequently replaced by an outpatient setting (with limited use of all quantifiable sleep parameters and with losing full possibility of studies on sleep architecture), most of recent data is not comparable. The heterogeneity of methodologies also precluded a meta-analysis of the results at this point.

## CONCLUSION

The majority of the studies found were class II or III of evidence and the number of randomized, double-blind clinical trials on sleep architecture was quite low. This could be related with the difficulty of assessing PSG in the laboratory. The exclusion of studies performed in children limited our conclusions, although sleep structure in children and their lifestyle have very different features in comparison to adult population. Thus, the methodologies we found were quite heterogeneous, which limited the establishment of conclusions and made impossible to do a meta-analysis.

However, the classic AEDs appear to have a more recognized effect on the structure of sleep, while the newer ones appear not to alter or having less interference on the structure of sleep. Future controlled studies with similar approaches and methodologies among diverse groups to test AEDs influence will be needed to establish more significant conclusions about the impact of this group of drugs, widely used in various pathologies, on sleep architecture. Research regarding the effect of the newer AEDs on sleep architecture parameters that have not class I of evidence articles, such as perampanel, etosuximide or without any evidence at all, such as brivaracetam, eslicarbazepine acetate, oxcarbazepine are even more needed.
